# Dexmedetomidine Improves Non-rapid Eye Movement Stage 2 Sleep in Children in the Intensive Care Unit on the First Night After Laparoscopic Surgery

**DOI:** 10.3389/fped.2022.871809

**Published:** 2022-04-27

**Authors:** Xian Zhang, Li Chang, Shou-Dong Pan, Fu-Xia Yan

**Affiliations:** ^1^Department of Anesthesiology, Capital Institute of Pediatrics Affiliated Children’s Hospital, Chinese Academy of Medical Sciences/Peking Union Medical College, Beijing, China; ^2^Department of Respiratory Medicine, Capital Institute of Pediatrics Affiliated Children’s Hospital, Beijing, China; ^3^Department of Anesthesiology, Capital Institute of Pediatrics Affiliated Children’s Hospital, Beijing, China; ^4^Department of Anesthesiology, Fuwai Hospital, Chinese Academy of Medical Sciences/Peking Union Medical College, Beijing, China

**Keywords:** children, sleep, dexmedetomidine, post-operative analgesia, sufentanil

## Abstract

**Background:**

Previous studies have reported that children who were admitted to the ICU experienced a significant decrease in sleep quality compared to home. We investigated the effects of dexmedetomidine as an adjunct to sufentanil on the sleep in children admitted to the ICU on the first night after major surgery.

**Methods:**

This is a prospective study From January to February 2022. Clinical trial number: ChiCTR2200055768, http://www.chictr.org.cn. Fifty-four children aged 1–10 years old children undergoing major laparoscopic surgery were recruited and randomly assigned to either the DEX group, in which intravenous dexmedetomidine (0.3 ug/kg/h) and sufentanil (0.04 ug/kg/h) were continuously infused intravenously for post-operative analgesia; or the SUF group, in which only sufentanil (0.04 ug/kg/h) was continuously infused. Patients were monitored with polysomnography (PSG) on the first night after surgery for 12 h. PSG, sleep architecture, physiologic variables and any types of side effects related to anesthesia and analgesia were recorded. The differences between the two groups were assessed using the chi-square and Wilcoxon rank-sum tests.

**Results:**

Fifty-four children completed data collection, of which thirty-four were 1–6 years old and twenty were aged >6 years. Compared to the SUF group, subjects in the DEX group aged 1–6 years displayed increased stage 2 sleep duration (*P* = 0.02) and light sleep duration (*P* = 0.02). Subjects aged >6 years in the DEX group also displayed increased stage 2 sleep duration (*P* = 0.035) and light sleep duration (*P* = 0.018), but decreased REM sleep percentage (*P* = 0). Additionally, the heart rate and blood pressure results differed between age groups, with the heart rates of subjects aged >6 years in DEX group decreasing at most time points compared to SUF group (*P* < 0.05).

**Conclusion:**

Dexmedetomidine prolonged N2 sleep and light sleep duration in the pediatric ICU after surgery but had different effects on the heart rate and blood pressure of subjects in different age groups.

## Introduction

Post-operative sleep disturbance in children admitted to the intensive care unit (ICU) has been previously described (1–3). The occurrence of sleep disorders can result in significant adverse outcomes, such as delirium, cardiovascular events, impaired immune function, prolonged mechanical ventilation, and post-operative decline in physical and mental health.

Dexmedetomidine (DEX) is a pharmacologically active dextroisomer of medetomidine and is a selective and specific α_2_-adrenoceptor agonist (4). Unlike other anesthetics, DEX induces a qualitative sedative response similar to natural sleep, an effect that is not seen in any other clinical sedative (5). It has been increasingly used for post-operative sedation because of this special effect (6).

Few studies have been conducted on the effects of DEX on sleep architecture in pediatric patients (7–9). The aim of this study was to investigate the effectiveness of DEX on the sleep architecture of non-mechanically ventilated children who underwent major laparoscopic surgery and who were transferred to the SICU post-operatively.

## Materials and Methods

### Patient Data

The study was approved by the Clinical Research Ethics Committee of the Capital Institute of Pediatrics (Beijing, China) and registered in the Chinese Clinical Trial Registry (ChiCTR2200055768). The parents or legal guardians of each child signed an informed consent form before surgery.

This was a randomized, double-blind controlled pilot study. The following inclusion criteria were used in this study: children aged 1–10 years; scheduled for elective major laparoscopic surgery (laparoscopic excision of choledochal cyst and laparoscopic-assisted pull-through operation for Hirschsprung’s disease) at Children’s Hospital, Capital Institute of Pediatrics (Beijing, China) from January to February 2022; had an American Society of Anesthesiologists physical status (ASA) value of I–II and planned to be transferred to the surgical intensive care unit (SICU) after surgery before 4:00 p.m. Patients with hepatic or renal dysfunction, epilepsy or abnormal EEG results, history of sedative drugs in the last month, obstructive sleep apnea syndrome, and those who required mechanical ventilation post-operatively were excluded from the study.

### Anesthesia Management

Propofol 2 mg/kg, rocuronium 0.6 mg/kg, and sufentanil 0.1 ug/kg were used for the induction of anesthesia. The anesthesia was maintained with propofol 10 mg/kg/h and remifentanil 0.3 ug/kg/h. Intraoperative monitoring of vital signs and fluid management were performed under uniform standards. The children were randomly assigned before induction to one of the following two experimental groups: (1) DEX group, in which a combination of DEX 0.3 ug/kg/h and sufentanil 0.04 ug/kg/h were continuously infused intravenously for post-operative analgesia; and (2) SUF group, in which only sufentanil 0.04 ug/kg/h was continuously infused intravenously for post-operative analgesia. The post-operative analgesia drugs were diluted in normal saline solution to 100 mL and added to an automatic electronic analgesia pump (AiPeng ZZB-I-50, Nantong, China) programmed to deliver 2 mL/h for 50 h.

Once transferred to the SICU, the children were monitored by electrocardiogram, invasive or non-invasive blood pressure, and pulse oxygen saturation continuously until they were transferred to the normal ward. Post-operative analgesia complications, including respiratory depression, hypoxemia, bradycardia, hypotension, nausea, and vomiting were treated by the SICU physician, as indicated.

### Polysomnography Monitoring

Polysomnography (PSG) was performed using a SONMO watch and an electroencephalography (EEG) Recording System 6 (SOMNO medics GmbH) from 8:00 p.m. the first night after surgery until 8:00 a.m. the following morning. The PSG included a four-channel electroencephalogram (F3/M2, F4/M1, C3/M2, and C4/M1), two-channel electrooculogram (E1/M2 and E2/M1), and one-channel chin electromyogram (Chin1–Chin2). The data were processed automatically according to the American Academy of Sleep Medicine Manual (10) and stored on a computer disk. The patient’s sleep architecture was scored using the DOMINO light software pediatric pattern (epoch by epoch) with standard criteria (10) and reviewed by a qualified sleep physician who was blinded to the study protocol and did not participate in patient care and data collection. The DOMINO light software of the SOMNO watch™ EEG system (SOMNO medics, Randersacker, Germany) has been proven to be a reliable tool for sleep analysis in a previous study (11). Sleep architecture was divided into wakefulness, rapid eye movement (REM) sleep, and non-rapid eye movement (NREM) sleep. NREM sleep was divided into three stages: N1, N2, and N3. In this work, light sleep refers to stages N1 and N2 and deep sleep refers to stage N3.

### Outcomes

The main outcome of this study was the characterization of sleep architecture during the first night after surgery, which included durations and percentages of each sleep stage (total sleep time, light sleep, deep sleep, and rapid eye movement sleep REM). We also evaluated heart rate (HR), blood pressure, respiratory rate (RR), and pulse oxygen saturation (SpO_2_) every hour, from 8:00 p.m. to 7:00 a.m. the next morning. The incidence of complications related to the post-operative analgesia were recorded, including respiratory depression (spontaneous respiratory rate less than 10 breaths per minute), hypoxemia (SpO_2_ less than 90% lasting more than one minute) (12), bradycardia (20% below the age-predicted or pre-operative baseline heart rate), hypotension [mild, presenting systolic arterial pressure (SAP) of 61–70 mmHg and mean arterial pressure (MAP) of 41–50 mmHg; moderate, presenting SAP of 51–60 mmHg and MAP of 31–40 mmHg; and severe, presenting SAP < 51 mmHg, MAP < 31 mmHg, lasting > 3 min] (13),over-sedation (difficult being woken up loudly or patted at 8 a.m. next morning), nausea, and vomiting.

### Statistical Analysis

IBM SPSS 21.0 software package was used to perform the statistical analysis. All descriptive values are presented as mean with standard deviation (mean ± SD), median with interquartile range (IQR), or frequency and percentage, as deemed appropriate. Comparisons between normal continuous data were performed using the Student’s *t* test and comparisons between non-normal continuous data were performed out using the Wilcoxon rank-sum test. Categorical data were analyzed using the chi-square or Fisher exact test. All tests were two-sided and *P* values < 0.05 were considered statistically significant.

N2 sleep duration was selected as the primary measure since DEX mainly affects NREM sleep (14). Based on the literature, we assume an expected increase in N2 duration with DEX + SUF for post-operative analgesia. Given a power of 0.9 and a type-1 error (alpha) of 0.05, a sample size of 54 surgical patients was required.

## Results

Seventy-seven children were screened for eligibility and fifty-nine of them were recruited for the study. During the observational period, PSG monitoring failed in three children due to the detachment of the electrode. Two patients were excluded from the sleep architecture analysis because signal interference rendered the polysomnographic data unanalyzable. In total, fifty-four patient records were analyzed for this study.

Due to the different sleep patterns among different ages, we grouped the patients by age into two groups when analyzing the results. A total of thirty-four patients were analyzed in the 1–6-year-old group (DEX = 16, SUF = 18), and twenty patients in the >6-year-old group (DEX = 11, SUF = 9).

### Demographic Data

There were no significant differences in the demographic data between the DEX and SUF group in patients aged 1–6 years (Table 1) and in patients aged >6-years (Table 2), including sex, age, weight, height, and type and duration of surgery.

**TABLE 1:** Demographic characteristics of patients aged 1–6 years old in the SUF and DEX experimental groups.

	DEX (*n* = 16)	SUF (*n* = 18)	*χ^2^/t*
Age (yrs)	2.6 ± 1.3	2.7 ± 1.1	1.66
Female, *n* (%)	8 (50%)	9 (50%)	0.63
Height (cm)	87.9 ± 12.3	87.9 ± 12.3	1.29
Weight (kg)	12.4 ± 3.4	12.4 ± 3.4	1.55
Surgery type	6/10	7/11	0.61
Surgery duration	218 ± 66	196 ± 47	0.25

*Type of surgery: Laparoscopic choledochal cyst excision/Laparoscopic-assisted pull-through operation for Hirschsprung’s disease.*

**TABLE 2:** Demographic characteristics of patients aged >6 years old in the SUF and DEX experimental groups.

	DEX (*n* = 11)	SUF (*n* = 9)	*χ^2^/t*
Age (yrs)	9.7 ± 2.9	9.2 ± 2.1	1.67
Female, *n* (%)	5 (45%)	5 (55%)	0.5
Height (cm)	129.8 ± 25.1	133.4 ± 14.7	0.74
Weight (kg)	26.7 ± 9.2	29.6 ± 10.1	0.012
Surgery type	4/7	5/4	0.34
Surgery duration	236 ± 69	205 ± 57	0.28

*Type of surgery: Laparoscopic choledochal cyst excision/Laparoscopic-assisted pull-through operation for Hirschsprung’s disease.*

#### Sleep Stage in 1–6-Year-Old Group

**FIGURE 1:**
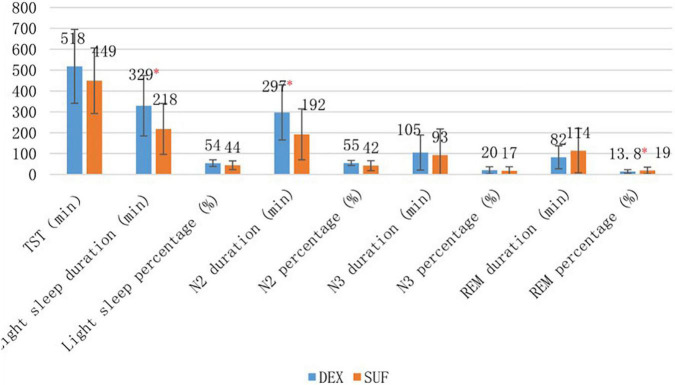
Sleep stages of patients aged 1–6 years old in the SUF and DEX experimental groups. *P < 0.05, from the comparison between the SUF and DEX groups.

**FIGURE 2:**
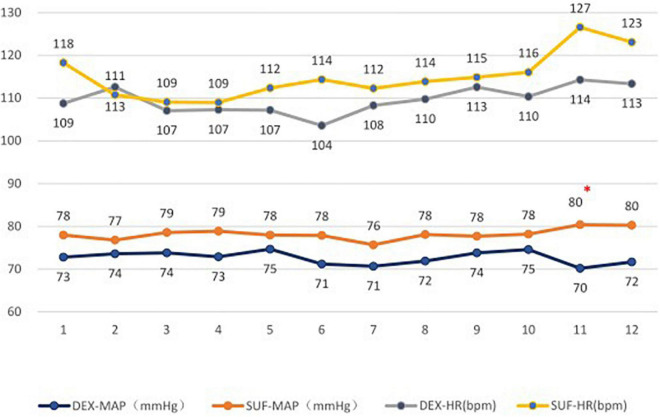
Heart rates (HR) and mean arterial pressure (MAP) in patients aged 1–6 years old in the SUF and DEX experimental groups. *P < 0.05, from the comparison between the SUF and DEX groups.

In the 1–6-year-old group, the duration of light sleep (329 ± 145 min vs. 217 ± 122 min, *P* = 0.02) and duration of stage N2 sleep (297 ± 132 min vs. 192 ± 122 min, *P* = 0.02) increased significantly in the DEX group compared to the SUF group (Figure 1). The difference in HR and MAP between the groups in the >6-year-old group were insignificant (Figure 2).

#### Sleep Stage in >6-Year-Old Group

**FIGURE 3:**
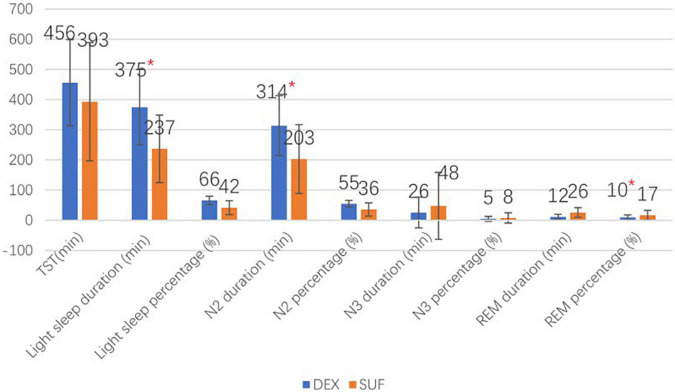
Sleep stages of patients aged >6 years old in the SUF and DEX experimental groups. *P < 0.05, from the comparison between the SUF and DEX groups.

**FIGURE 4:**
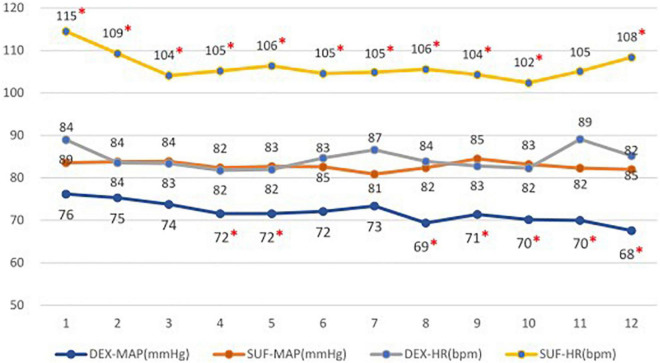
Heart rates (HR) and mean arterial pressure (MAP) in patients aged >6 years old in the SUF and DEX experimental groups. *P < 0.05, from the comparison between the SUF and DEX groups.

In the >6-year-old group, the duration of light sleep (375 ± 125 min vs. 237 ± 113 min, *P* = 0.018) and duration of stage N2 sleep (314 ± 99 min vs. 203 ± 114 min, *P* = 0.035) also increased significantly in the DEX group compared to the SUF group. Additionally, there was a significant decrease in the percentage of the REM sleep stage (12.5 ± 8.3% vs. 25.7 ± 16%, *P* = 0.043) in the DEX group compared to the SUF group (Figure 3). Noticeable differences in HR and blood pressure were observed at almost all observation points (Figure 4).

## Discussion

Our study was to investigate the effectiveness of DEX on the sleep architecture of non-mechanically ventilated children who underwent major laparoscopic surgery and who were transferred to the SICU post-operatively. According to the comparison after age grouping, the light sleep and N2 sleep duration of children using DEX + SUF were prolonged. These results are consistent with previously reported results of patients in the ICU (15–18) and in surgical patients (19–23). These effects are likely related to the DEX-mediated sedation as an α_2_ agonist, which converges to an endogenous NREM sleep-promoting pathway (4). DEX sedatives activate sleep pathways that work on nuclei upstream of the ventrolateral preoptic nucleus (VLPO) at the level of the locus coeruleus (LC) and thus may produce more restorative sleep (4).

In a study that involved the intraoperative use of DEX in children aged 2–10 years, they found that HR was statistically lower during PACU in the DEX group (24). The study of EEG examination of autistic children aged 2–11 years under DEX sedation found that the heart rate and blood pressure of all children decreased compared with the baseline (25). Our study found that DEX administration caused a significant reduction in HR and blood pressure at almost all observation points in >6-years group. Among children older than 6 years old, the HR of 2 patients dropped to less than 60 bpm in the DEX group, with a minimum HR observed of 52 bpm, and the administration of atropine 0.01 mg/kg was able to effectively increase HR. This may be related to the sympatholytic properties of DEX. But in our study, we also found that DEX had little effect on HR and blood pressure during sleep with children aged 1–6 years. We considered that this result might be related to sympathetic nervous system hypoplasia in younger children. Dose-related Hypotension and bradycardia are more frequent adverse events associated with DEX (24, 26). The dose of DEX (more than 0.5 ug/kg/h) (24, 26) is associated with the incidence of adverse effects (27), and a lower-dose (0.1 ug/kg/h) may not sufficient to produce effects on sleep architecture (19). We applied a moderate DEX dose (0.3 ug/kg/h) in ours study. None of the patients in this study developed respiratory depression, delirium, or over-sedation. For younger children, this dose of DEX may effectively prolong N2 sleep without causing its sympatholytic effect. However, adverse reactions from DEX administration differ in children of varying ages. More studies may be needed to confirm the dose and causes of adverse reactions in children of different ages. As such, caution should be used when administrating DEX in children.

A study of healthy volunteers found that, DEX promotes biomimetic non-rapid eye movement stage 3 sleep in human (28). However, there is no effect of DEX on stage N3 was observed in our study. It may be related to the difference in the subject population. One group is healthy volunteers (29), and the other is pediatric patients after surgery. Previous studies have reported that N3 stage and REM sleep are severely or completely suppressed on the first night after major open abdominal surgery in adult patients (19, 30). The degree of sleep disturbance in children appears to be less severe than that reported in adults (17). In our study, N3 stage was absent in only eight patients (15.1%), and REM sleep was absent in only one patient (1.9%). The pediatric population may have longer N3 stage and REM sleep than adults due to the physiological requirements for their development (8). Surgeries, comorbidities and the use of medications can also lead to the development of post-operative sleep disturbance (19, 31, 32). The effect of DEX on sleep in different populations may need to be confirmed by more studies.

This study had some limitations worth mentioning. First, the fixation of a head electrode caused discomfort in some children. Second, the sample size of this study was relatively small and further large-scale studies are required to clarify the risks and the appropriate dose of DEX to be administered in pediatric patients. Third, no baseline sleep study was performed. We evaluated the pre-operative sleep quality of the patients by asking the parents or legal guardians about the medical history of the patients and sleep disturbance was not common in the study population. Lastly, since PSG monitoring was performed from 8:00 p.m. to 8:00 a.m. on the first night after surgery and all the patients were sent to the SICU before 4:00 p.m., the sleep stages before 8:00 p.m. were not included. To minimize the residual effects of anesthesia on the results, the same intraoperative analgesic regimen was used in this study. We did not include any objective evaluations on sleep quality and pain because of the particularity of the pediatric population and the duration of observation during the night.

## Conclusion

Our study showed that in non-mechanically ventilated children aged 1–10 years who were admitted to the SICU after major laparoscopic surgery, a moderate dose of DEX infusion (0.3 ug/kg/h) improved stage N2 sleep. However, this infusion resulted in a potential risk of hypotension and bradycardia in children aged older than 6 years and more careful monitoring was required.

## Data Availability Statement

The original contributions presented in the study are included in the article/supplementary material, further inquiries can be directed to the corresponding authors.

## Ethics Statement

The studies involving human participants were reviewed and approved by Ethics Committee of Capital Institute of Pediatrics. Written informed consent to participate in this study was provided by the participants’ legal guardian/next of kin.

## Author Contributions

XZ drafted the initial manuscript. LC reviewed DOMINO light software pediatric pattern epoch by epoch, diagnosed the patients, and also reviewed and revised the manuscript. S-DP was a consultant of anesthesia, analyzed data, and reviewed and revised the manuscript. F-XY was the supervisor of anesthesia, guided research design, and reviewed and revised the manuscript. All authors approved the final manuscript as submitted and agreed to be accountable for all aspects of the work.

## Conflict of Interest

The authors declare that the research was conducted in the absence of any commercial or financial relationships that could be construed as a potential conflict of interest.

## Publisher’s Note

All claims expressed in this article are solely those of the authors and do not necessarily represent those of their affiliated organizations, or those of the publisher, the editors and the reviewers. Any product that may be evaluated in this article, or claim that may be made by its manufacturer, is not guaranteed or endorsed by the publisher.
